# In Memorium: Herman P. Schwan [1915–2005]

**DOI:** 10.1186/1475-925X-4-21

**Published:** 2005-03-25

**Authors:** Kenneth R Foster

**Affiliations:** 1 Department of Bioengineering, University of Pennsylvania, 220 South 33rd Street,, Philadelphia, PA 19104, USA

## Abstract

Herman P. Schwan [1915–2005] was a distinguished scientist and engineer, and a founding father of the field of biomedical engineering. A man of integrity, Schwan influenced the lives of many, including his wife and children, and his many students and colleagues. Active in science until nearly the end of his life, he will be very much missed by his family and many colleagues.

Herman Paul Schwan, renowned scientist and engineer, loving and devoted husband and father, died quietly in his home in Radnor, Pennsylvania on March 17, 2005. Herman P. Schwan was born in Aachen, Germany in 1915. He obtained the German superior school certificate with distinction in Goettingen, 1934. He studied mathematics, physics, and engineering in Goettingen and then biophysics in Frankfurt. He obtained his Ph.D. in biophysics at the University of Frankfurt in 1940 with distinction, his teaching certificate at the University and his professional doctorate (Dr. habil) in the fields of physics and biophysics in 1946.

Schwan worked in 1936–37 and again in 1938 with Telefunken on high frequency and microwave measuring techniques. He became a research associate with the Max Planck Institute of Biophysics in Frankfurt in 1937, an assistant professor with the University of Frankfurt and associate director of the Max Planck Institute in 1946. In 1947 he came to the United States, working at the Aeromedical Equipment Laboratory of the U.S. Naval Base in Philadelphia. He joined the University of Pennsylvania in 1950. In 1952 he was appointed Head of the Electromedical Division of the Moore School and in 1961 Chairman of the Graduate School of Arts and Sciences Group on Biomedical Electronic Engineering. In 1972 he became Chairman of the Bioengineering Department. He retired as the Alfred Fitler Moore Professor Emeritus in 1983.

Over the course of his long career, Schwan published more than 300 scientific papers and gave countless lectures. He received numerous awards in recognition of his contributions, including the Edison Medal of the IEEE and the first d'Arsonval Award of the Bioelectromagnetics Society, membership in the National Academy of Engineering, and several honorary degrees. An extended biography of Schwan was published recently in the Annual Reviews of Biomedical Engineering [1].

As a scientist, Schwan is best known for many biophysical studies related to electrical properties of cells and tissues, and on nonthermal mechanisms of interaction of fields with biological systems. He discovered or provided important theoretical insights into phenomena such as the large low-frequency dielectric dispersion that is found in biological material, and electrically induced forces on cells.

Schwan was also deeply involved in the issue of possible health effects of nonionizing electromagnetic fields. His letter to the U.S. Navy in 1953, proposing a safe limit for human exposure to microwave energy of 100 W/m^2^ (based on thermal analysis) became the basis for exposure standards in the U.S. and elsewhere. Among his many other committee activities in this field, he chaired the committee that established the first (1965) U.S. exposure limit for radiofrequency energy, for the American National Standards Institute. This standard evolved into the present IEEE C95.1 standard and was widely influential in the development of exposure limits around the world.

Schwan was married in 1949 to Anne Marie Del Borrello of Philadelphia. In addition to his wife, Herman is survived by five children and six grandchildren. He was a mentor to all of them, first and foremost teaching them always to think for themselves and never to just follow the crowd. A man of integrity, Schwan influenced the lives of many, including his wife and children, and his many students and colleagues.
